# Mapping Rural–Urban Disparities in Otolaryngology Access: A Statewide Analysis of Georgia's ENT Workforce

**DOI:** 10.1002/oto2.70286

**Published:** 2026-08-04

**Authors:** Shervin Eskandari, William E. Long, Benjamin Aderinwale, Dev Shah, Kush Patel, Lilian Ballesteros, Camilo Reyes

**Affiliations:** ^1^ Medical College of Georgia Augusta University Augusta Georgia USA; ^2^ Department of Otolaryngology‐Head and Neck Surgery University of Mississippi Medical Center Jackson Mississippi USA; ^3^ Department of Otolaryngology‐Head and Neck Surgery Emory University School of Medicine Atlanta Georgia USA

**Keywords:** geographic disparities, healthcare access, Otolaryngology workforce, physician distribution, rural health

## Abstract

**Objective:**

While healthcare disparities between rural and urban populations have been documented, access to subspecialty care in rural communities remains poorly characterized. This study aims to quantify rural‐urban disparities regarding access to otolaryngologists in Georgia.

**Study Design:**

Cross‐sectional.

**Setting:**

Center for Medicare and Medicaid Services (CMS), Georgia Composite Medical Board (GCMB), and American Academy of Otolaryngology‐Head and Neck Surgery (AAO‐HNS) databases.

**Methods:**

The CMS and GCMB databases were used to identify otolaryngologists in Georgia and their primary office location. Residency and fellowship training information was collected through the AAO‐HNS database and institutional websites. County‐level demographic data were obtained from the 2020 US Census. The 2023 Rural‐Urban Continuum Codes were used to classify metropolitan (1‐3) and nonmetropolitan (4‐9) counties.

**Results:**

A total of 367 otolaryngologists were identified across Georgia's 159 counties (74 metropolitan, 85 nonmetropolitan). Common residency training states included Georgia (20.4%) and New York (6.8%). Overall, 42.5% of otolaryngologists completed fellowship training, with the most common being pediatric otolaryngology (23.7%) and facial plastics (21.8%). Metropolitan counties had an average travel distance of 10.23 miles to the nearest otolaryngologist and 1.91 otolaryngologists per 100,000 residents, compared to 20.32 miles and 0.76 otolaryngologists per 100,000 residents in nonmetropolitan counties (*P* < .001). Most otolaryngologists (n = 232, 63.2%) were in counties classified as code 1 (most urbanized), which also had the lowest travel burden to the nearest otolaryngologist. Additionally, 12.8% of otolaryngologists practiced in academic settings.

**Conclusion:**

There are significant rural‐urban ENT‐related disparities in the state of Georgia, with a shortage of otolaryngologists in rural counties.

Geographic healthcare disparities persist in the United States, with rural residents having less access to subspecialty care than urban populations.^1^ Among medical specialties, otolaryngologists are particularly concentrated in urban areas.^2^, ^3^ This imbalance is especially concerning given the broad scope of otolaryngologic practice, encompassing many of the most prevalent conditions for which rural patients seek care.^4^, ^5^ While primary care physicians may treat uncomplicated ear, nose, and throat (ENT) issues,^6^, ^7^ expert surgical care is needed for more complex cases, and residents of rural areas often have limited access to this subspecialty care.^8^, ^9^, ^10^

The notion that rural residents face reduced access to care is not new; a recent study by Winters et al found significantly fewer otolaryngologists per capita in rural counties of seven southeastern states, along with higher populations of uninsured patients in many of these areas.^11^ With 25.9% of its 10.7 million residents in rural areas, Georgia has the nation's fifth‐largest rural population, highlighting the need to investigate this disparity.^12^, ^13^ Additionally, Georgia struggles with a well‐described physician shortage, and ranks 40th among states nationally in physicians per 10,000 residents according to data analyzed by the Centers for Disease Control and Prevention.^14^ Despite these challenges, data on the geographic distribution of otolaryngologists in Georgia remains limited.

Improving rural health is multifaceted, and expanding access to otolaryngologic care plays a meaningful role. Rural residents have higher tobacco use and lower awareness of human papillomavirus—both risk factors for head and neck cancer.^15^, ^16^, ^17^ Increased distance from specialty care is also associated with worse outcomes across several ENT‐related conditions.^18^, ^19^, ^20^, ^21^, ^22^, ^23^, ^24^ Policy decisions, resource allocation, and targeted interventions aimed to address these problems require an understanding of the geospatial distribution of the otolaryngology workforce. Thus, our study aimed to map the distribution of otolaryngologists in Georgia and quantify access disparities between urban and rural areas.

## Methods

Expanding on previous workforce analyses,^8^, ^9^, ^10^, ^25^, ^26^, ^27^ a cross‐sectional study was performed to determine the distribution of practicing otolaryngologists in the state of Georgia. The 2023 Centers for Medicare and Medicaid Services (CMS) database,^28^ which includes a summary of services and procedures to Medicare Part B beneficiaries, was used to identify an initial list of otolaryngologists in Georgia. Additionally, the Georgia Composite Medical Board (GCMB) database,^29^ maintains an updated directory of all healthcare professionals in the state. This database was used to cross‐reference the CMS database and to obtain information for any additional otolaryngologists practicing in Georgia along with their primary office location. Information regarding each surgeon's state of residency and fellowship training was collected through the American Academy of Otolaryngology‐Head and Neck Surgery (AAO‐HNS) database,^30^ and institutional websites when applicable.

After aggregating data across sources, a unique roster of practicing otolaryngologists in Georgia that included state of residency training, practice site, fellowship training, and demographic variables was compiled. Practice setting was categorized as academic (hospital with an affiliated residency program) or non‐academic. Geographic assignment was based on the primary practice location reported in the GCMB database, with locations manually verified through review of institutional and practice websites to ensure accuracy. Additionally, surgeons with inactive or lapsed licenses were excluded, and only actively licensed surgeons were included for analysis. Residents and fellows in training were also excluded from analysis.

County‐level data including population, median household income, and land area in square miles, were obtained from the US Census.^31^, ^32^ Rurality was additionally represented as a function of the percent‐rural population, as obtained from the 2020 US Census data.^12^ To classify counties as metropolitan (metro) or nonmetropolitan (nonmetro), the 2023 Rural‐Urban Continuum Codes (RUCCs) were utilized.^33^ These codes differentiate metro counties based on the population size of their metropolitan area, while categorizing nonmetro counties based on their level of urbanization and proximity to a metro area. This classification system was selected for its ability to capture both population size and proximity to metropolitan areas, allowing for a more nuanced classification of rurality. The classification system consists of three metro and six nonmetro categories, assigning each county one of nine possible codes. Metro counties fall into one of three groups: (1) those within metro areas of at least 1 million residents, (2) those in metro areas with populations between 250,000 and 1 million, and (3) those in metro areas with fewer than 250,000 residents. Nonmetro counties are further categorized as follows: (4) urban areas with 20,000 or more residents adjacent to a metro area, (5) urban areas with 20,000 or more residents not adjacent to a metro area, (6) urban areas with populations between 5000 and 20,000 that are adjacent to a metro area, (7) urban areas of 5000 to 20,000 residents that are not adjacent to a metro area, (8) areas with fewer than 5000 residents near a metro area, and (9) areas with fewer than 5000 residents that are not near a metro area.

The number of otolaryngologists per 100,000 residents was calculated for each county. Additionally, the number of otolaryngologists per 100 square miles was calculated for each county. To assess travel burden, the linear distance from the geographic centroid of each county to the nearest otolaryngology practice was calculated.

Statistical analyses were conducted using SPSS v29.0.0.0 (IBM). The Kruskal‐Wallis test was used to compare data between the nine RUCCs, the Mann‐Whitney *U* test was used to compare data between metro and nonmetro counties, and the chi‐square test was used to compare the proportion of fellowship‐trained surgeons between metropolitan and nonmetropolitan counties. Statistical significance was defined as *P* < .05.

This study used publicly available, aggregate data and did not meet the criteria for Human Subjects Research requiring approval from the Augusta University Institutional Review Board.

## Results

### Otolaryngologist Demographic Data

A total of 367 practicing otolaryngologists were identified across Georgia, with 95.4% holding a Doctor of Medicine (MD) degree. The most common states of residency training included Georgia (20.4%), New York (6.8%), and North Carolina (5.4%), and 4.9% of physicians trained internationally. Additionally, 42.5% of physicians were fellowship‐trained, with most common fellowship types including pediatric otolaryngology (23.7%), facial plastic and reconstructive surgery (21.8%), and head and neck (19.9%). Regarding practice setting, 12.8% of physicians practiced in an academic setting. Detailed information regarding otolaryngologist demographic data is displayed in Table 1.

**Table 1:** Demographics and Practice Setting of Otolaryngologists in Georgia

State of residency training	(n = 367)
Georgia	75 (20.4%)
New York	25 (6.8%)
North Carolina	20 (5.4%)
California	18 (4.9%)
Virginia	17 (4.6%)
Other US States	194 (52.9%)
International	18 (4.9%)

Fellowship type	(n = 156)
Pediatric Otolaryngology	37 (23.7%)
Facial Plastic and Reconstructive Surgery	34 (21.8%)
Head and Neck	31 (19.9%)
Otology/Neurotology	18 (11.5%)
Rhinology	16 (10.3%)
Laryngology	12 (7.7%)
Sleep Medicine	5 (3.2%)
Allergy and Immunology	3 (1.9%)

Practice setting	(n = 367)
Academic	47 (12.8%)
Non‐Academic	320 (87.2%)

### Geographic Characteristics and Otolaryngologist Distribution by RUCC

Georgia's 159 counties were distributed across all nine RUCCs, with a total population of 10,711,908 residents spanning 57,716 square miles. Of this population, 25.9% (n = 2,777,922) resided in rural areas. Counties classified as RUCC 1 had 12.10% of their residents living in rural areas, whereas counties classified as RUCCs 8 and 9 had 98.30% and 94.40% of their residents living in rural areas, respectively.

Of the 367 otolaryngologists across the state, most were concentrated in counties classified as RUCC 1 (232 otolaryngologists), and these counties had the lowest travel burden to the nearest physician compared to counties in any other RUCC (average travel burden = 7.10 miles). In contrast, counties designated as RUCCs 8 and 9 had only single otolaryngologists present. Detailed information regarding county demographics and physician distribution by RUCC is further depicted in Table 2.

**Table 2:** Data for Georgia Counties by Rural‐Urban Classification (RUCC)

RUCC	Counties	Total population	Rural Census	Total % Rural	Total square miles	Total no. ENT	Average ENT/100k*	Average ENT/100 square miles*	Average travel burden (miles)*
1	29	6,104,803	737,128	12.10%	9114	232	2.26 (2.34)	2.11 (4.00)	7.10 (7.54)
2	17	1,242,810	287,299	23.10%	6186	63	1.74 (3.35)	1.16 (2.17)	15.95 (9.77)
3	28	1,577,355	542,155	34.40%	10,221	48	1.65 (2.35)	0.66 (1.42)	9.98 (7.73)
4	9	520,764	259,398	49.80%	4337	11	2.19 (2.23)	0.24 (0.20)	7.19 (9.23)
5	2	77,595	28,405	36.60%	1160	0	‐	‐	15.03 (16.93)
6	16	426,970	255,040	59.70%	5730	6	1.15 (1.85)	0.10 (0.16)	15.83 (11.07)
7	8	195,225	118,359	60.60%	3749	5	1.96 (2.84)	0.12 (0.19)	16.49 (12.31)
8	31	391,092	384,610	98.30%	11,546	1	0.16 (0.91)	0.01 (0.05)	26.90 (10.10)
9	19	175,294	165,528	94.40%	5671	1	0.31 (1.36)	0.01 (0.06)	21.74 (10.45)
Total	159	10,711,908	2,777,922	25.90%	57,716	367	1.30 (2.24)	0.66 (2.07)	15.62 (11.78)

Data are presented as mean (standard deviation) when applicable.

Abbreviation: ENT, otolaryngologist.

*Statistically significant (*P* < .001).

### Geographic Characteristics and Otolaryngologist Distribution by Metropolitan Status

In total, 74 counties were classified as metropolitan and 85 were nonmetropolitan. Metropolitan counties contained 8,924,968 residents and nonmetropolitan counties contained 1,786,940 residents. On average nonmetropolitan counties had a significantly greater percentage of residents living in rural areas compared to metropolitan counties (mean [SD] % rural in metropolitan versus nonmetropolitan = 54.40 (36.10) vs 80.11 (23.20), *P* < .001). Additionally, metropolitan counties had a significantly greater median household income when compared to nonmetropolitan counties (mean [SD] median household income metropolitan vs nonmetropolitan = $70,153.81 ($18,908.06) vs $52,072.22 ($10,011.87), *P* < .001).

Metropolitan counties contained 93.5% (n = 343) of otolaryngologists, compared to only 6.5% (n = 24) of otolaryngologists across all nonmetropolitan counties. A total of 108 counties (67.9%) had no practicing otolaryngologist, representing 2,186,017 individuals, with a mean (SD) travel distance of 21.73 (9.19) miles to the nearest surgeon. Compared to nonmetropolitan counties, metropolitan counties had significantly greater otolaryngologists per 100k residents (mean [SD] = 1.91 (2.59) vs 0.76 (1.74), *P* < .001) and per 100 square miles (mean [SD] = 1.34 (2.89) vs 0.06 (0.14), *P* < .001). Additionally, 44.3% (152/343) of metropolitan surgeons had completed fellowship training, compared with 16.7% (4/24) in nonmetropolitan counties (*P* = .008).

Detailed results regarding county demographics and physician distribution by metropolitan status are depicted in Table 3.

**Table 3:** Otolaryngologist Access in Metropolitan and Nonmetropolitan Counties

	Metropolitan	Nonmetropolitan	*P* value
Number of counties	74	85	‐
Total population	8,924,968	1,786,940	‐
Total rural census	1,566,582	1,211,340	‐
Total % rural	17.60%	67.80%	‐
Total square miles	25,522	32,194	‐
Total ENT	343	24	‐
Average square miles	344.90 (130.98)	378.75 (166.84)	.443
Average % rural	54.40% (36.10)	80.11% (23.20)	<.001
Average median household income	$70,153.81 ($18,908.06)	$52,072.22 ($10,011.87)	<.001
Average number of ENT	4.64 (13.50)	0.28 (0.68)	<.001
Average ENT/100k	1.91 (2.59)	0.76 (1.74)	<.001
Average ENT/100 square miles	1.34 (2.89)	0.06 (0.14)	<.001
Average travel burden (miles)	10.23 (8.74)	20.32 (12.11)	<.001

Data are presented as mean (standard deviation) when applicable.

Abbreviation: ENT, otolaryngologist.

**Figure 1:**
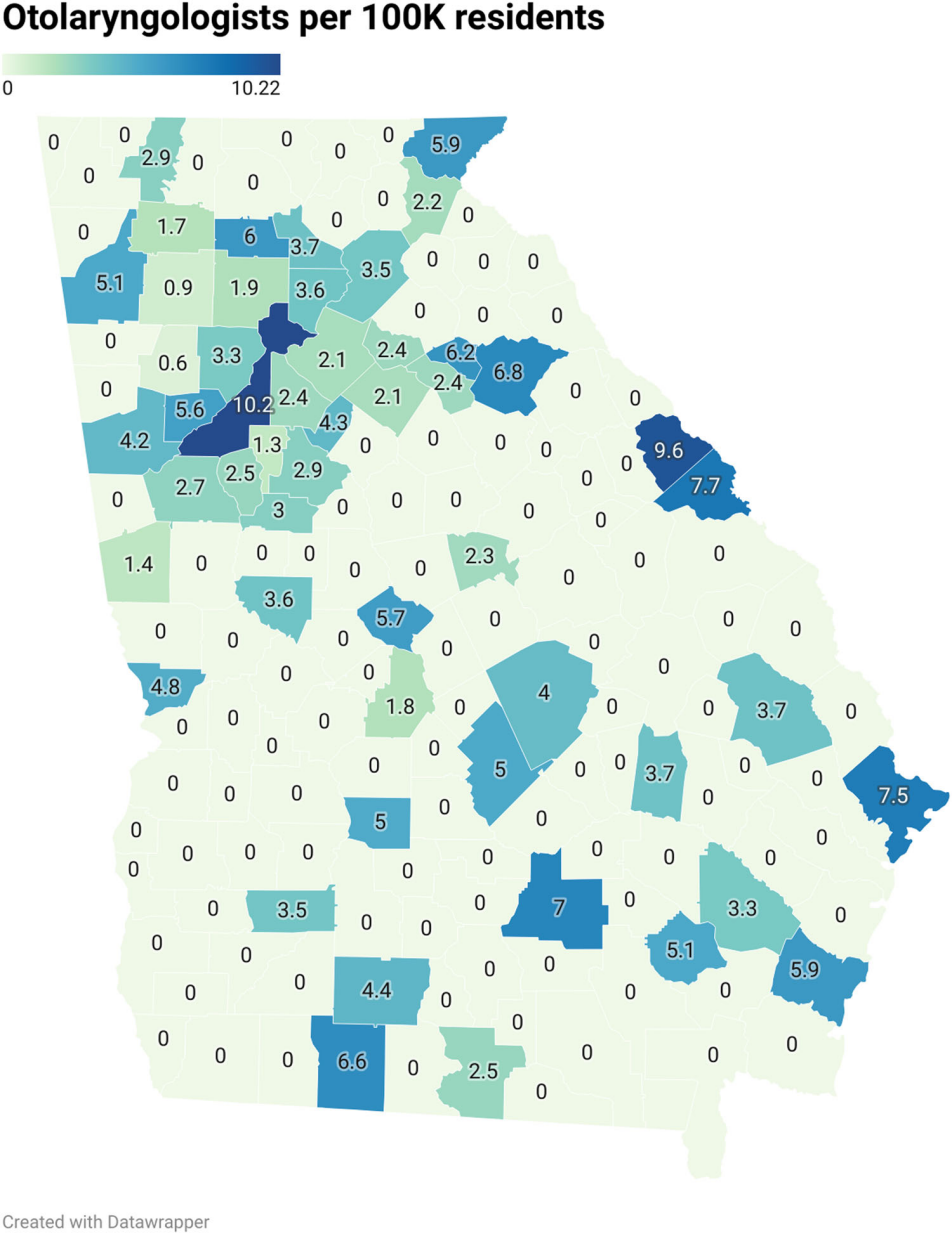
Distribution of the Georgia otolaryngology workforce as represented by the number of otolaryngologists per 100,000 people.

**Figure 2:**
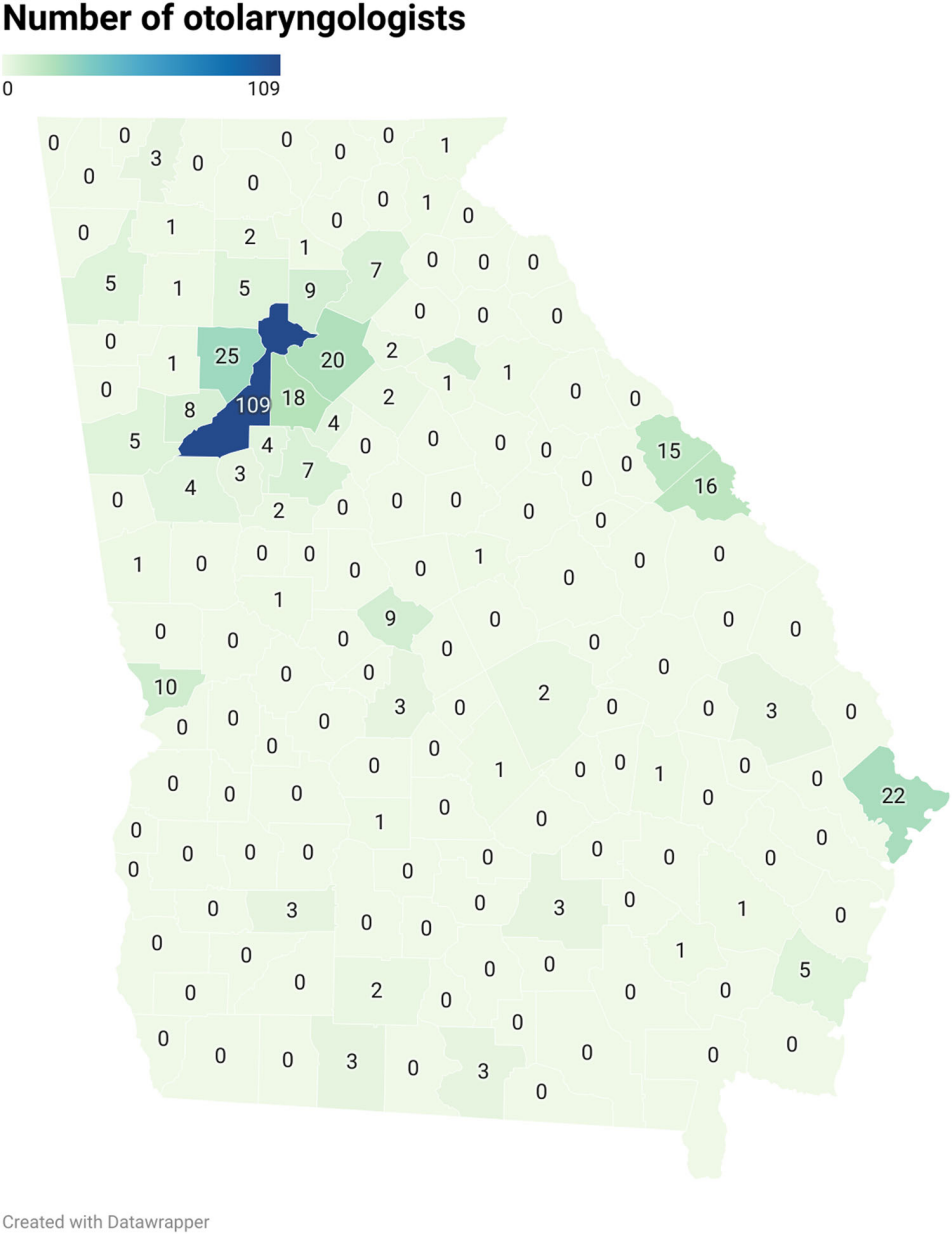
Distribution of the Georgia otolaryngology workforce as represented by the number of otolaryngologists per county.

Residents in nonmetropolitan counties also had significantly greater travel burdens to the nearest otolaryngologist compared to metropolitan counties (mean [SD] travel burden in miles = 20.32 (12.11) vs 10.23 (8.74), *P* < .001). The geographic distribution of these physicians is illustrated in Figures 1 and 2 as the number of physicians per 100k residents (Figure 1) and the number of physicians per county (Figure 2).

## Discussion

Otolaryngology–Head and Neck Surgery represents an important component of the physician workforce, providing specialized care across a broad spectrum of conditions. Georgia ranks as the eighth largest state by population and has the fifth highest rural population in the nation, with 25.9% of its population living in rural areas.^12^, ^13^ Given the high prevalence of ENT‐related conditions,^34^, ^35^ workforce planning and optimal resource allocation within Georgia are essential to ensure equitable access to ENT‐related care. While prior studies have explored various social determinants of health relevant to otolaryngology, research related to rural‐urban disparities remains sparse.^36^ Thus, our study aimed to better understand the distribution of the otolaryngology workforce across Georgia, supporting possible future labor redistribution interventions.

Our analysis revealed substantial geographic disparities in the distribution of the otolaryngology workforce, with 93.5% of otolaryngologists practicing in metropolitan counties. This imbalance closely reflects national patterns and even exceeds findings of a recent national reporting that 92.1% of otolaryngologists practice in urban areas.^2^, ^11^, ^37^ Among metro counties, Fulton and Richmond counties had among the highest number of otolaryngologists per 100,000 residents. These counties share a common characteristic—the presence of an academic medical center with affiliated medical schools and otolaryngology residencies, which play a role in attracting and retaining otolaryngologists within metro regions. Additionally, 63.2% of Georgia's otolaryngologists were in counties classified as RUCC 1, a proportion comparable to those reported in Florida and Pennsylvania (66.3% and 60.6%, respectively).^9^, ^25^

When stratified by metropolitan status, metro counties in Georgia had 1.91 otolaryngologists per 100,000 people and 1.34 otolaryngologists per 100 square miles. These values fall below those reported in comparable state‐level analyses from Pennsylvania (2.40/100k people) and Florida (2.87/100k people), but exceed the metropolitan density observed in Illinois (1.32/100k people).^8^, ^9^, ^25^ Conversely, nonmetro counties in Georgia had only 0.76 otolaryngologists per 100,000 people and 0.06 otolaryngologists per 100 square miles. These non‐metro rates are substantially lower than Pennsylvania (2.40/100k people), higher than Illinois (0.46/100k people), and comparable to Florida (0.76/100k people).^8^, ^9^, ^25^

Our analysis also showed that 42.5% of practicing otolaryngologists in Georgia had completed fellowship training, a proportion slightly higher than that reported in Florida (38.5%). This finding is particularly important given that not all otolaryngologists provide comprehensive general ENT care, and national data demonstrate an increasing trend toward fellowship training among residents, resulting in a workforce with a growing number of subspecialists.^38^, ^39^ In our cohort, fellowship‐trained surgeons were also predominantly concentrated in metropolitan counties, further limiting access to subspecialty care in nonmetropolitan regions. As clinical practice becomes more subspecialized, access to broad‐based otolaryngologic services may become further constrained in regions already experiencing provider shortages, an important consideration when evaluating workforce capacity. These trends highlight the need for workforce planning that considers not only geographic distribution but also subspecialization patterns, as increasing fellowship training may exacerbate access challenges in underserved regions.

The travel burden associated with accessing ENT care in rural Georgia counties was apparent in our study. Patients in nonmetro counties faced a mean travel distance of 20.32 miles to the nearest otolaryngologist, compared to 10.23 miles in metro counties (*P* < .001). Furthermore, this figure does not capture other barriers and logistical challenges present in rural environments including limitations of public transportation, roadway infrastructure, and internet access.^40^, ^41^, ^42^ In the context of these existing barriers, significant travel burdens can lead to delays in timely diagnosis and management leading to worse outcomes, especially for conditions that require multidisciplinary care, and longitudinal management and follow‐up. Additionally, 67.9% of counties lacked a practicing otolaryngologist, most of which were nonmetropolitan. This is particularly concerning given that median household incomes in nonmetro counties were, on average, $20,000 below the state median. The absence of otolaryngologists in these counties is not unexpected, however, as previous studies have reported poverty, lower socioeconomic status, and reduced educational attainment being correlated with a reduced density of otolaryngologists.^3^

To address the geographical disparity that rural communities face, several efforts have been employed, and programs designed to promote physicians practicing in rural areas are in place.^37^ Loan forgiveness programs and specialized rural health opportunities for medical students have also been implemented,^43^ but their effectiveness remains unclear. Strengthening the educational pipeline is also important. Georgia currently offers only two otolaryngology residency programs statewide. Expanding residency positions faces major barriers, as graduate medical education (GME) funding is capped, and new positions require substantial institutional financial support. These costs necessitate innovative solutions like state‐level GME expansion or public‐private partnerships. Without such targeted investment in residency growth, shortages in otolaryngology access may persist as demand continues to increase.

This study is not without its limitations. First, the cross‐sectional design provides a single time‐point assessment and cannot determine whether observed disparities are evolving over time. Second, access was defined using geographic proxies, including physician location, workforce density, and travel distance, which may not fully capture the multidimensional nature of healthcare access. Additionally, county‐level classification may obscure within‐county heterogeneity, particularly in geographically large regions. Our study also excluded advanced practice providers, audiologists, speech‐language pathologists, and other healthcare professionals. Furthermore, each otolaryngologist's associated county was recorded based on their primary location as provided by the GCMB. It is not uncommon for surgeons to work at multiple sites nearby their primary location, expanding their reach. Our study also did not include data regarding the availability of telemedicine. Additionally, to ensure consistency and reproducibility in geographic comparisons, travel burden was calculated using straight‐line (linear) distances rather than driving distances. While this approach simplifies analysis, it likely underestimates actual travel time between county centroids and ENT practices. Furthermore, the use of county centroids as a proxy for population location may introduce measurement error, particularly in sparsely populated counties where residents are distributed unevenly. As this study is limited to a single state, the findings may not be generalizable to other regions with differing demographic, geographic, and healthcare system characteristics. Future research should incorporate real‐world travel metrics and explore strategies to reduce these access gaps in rural communities.

## Conclusion

There are significant rural‐urban disparities in access to ENT care in the state of Georgia with a shortage of otolaryngologists in rural counties and the vast majority of providers concentrated in metropolitan counties. These gaps are compounded by increasing subspecialization within the workforce and substantial travel burdens for rural patients. Addressing these inequities will require targeted workforce planning, expansion of training opportunities, and innovative strategies to improve access in underserved regions.

## Author Contributions

**Shervin Eskandari**, conception, data collection, methodology, statistical analysis, manuscript writing, manuscript editing; **William E. Long:** data collection, statistical analysis, manuscript writing, manuscript editing; **Benjamin Aderinwale**, data collection, manuscript writing, manuscript editing; **Dev Shah**, data collection, manuscript writing, manuscript editing; **Kush Patel**, manuscript writing, manuscript editing; **Lilian Ballesteros,** manuscript writing, manuscript editing; **Camilo Reyes,** manuscript editing, supervision.

## Disclosures

### Competing interests

None.

### Funding source

None.

## References

[1] Barreto T , Jetty A , Eden AR , Petterson S , Bazemore A , Peterson LE . Distribution of physician specialties by rurality. J Rural Health. 2021;37(4):714‐722. 10.1111/jrh.12548 33274780

[2] Liu DH , Ge M , Smith SS , Park C , Ference EH . Geographic distribution of otolaryngology advance practice providers and physicians. Otolaryngol Head Neck Surg. 2022;167(1):48‐55. 10.1177/01945998211040408 34428088

[3] Gadkaree SK , McCarty JC , Siu J , et al. Variation in the geographic distribution of the otolaryngology workforce: a national geospatial analysis. Otolaryngol Head Neck Surg. 2020;162(5):649‐657. 10.1177/0194599820908860 32122214

[4] Emerson LP , Job A , Abraham V . A model for provision of ENT health care service at primary and secondary hospital level in a developing country. BioMed Res Int. 2013;2013:562643. 10.1155/2013/562643 24078919 PMC3776560

[5] Singh A , Kumar S . A survey of ear, nose and throat disorders in rural India. Indian J Otolaryngol Head Neck Surg. 2010;62(2):121‐124. 10.1007/s12070-010-0027-3 23120697 PMC3450296

[6] Sorichetti BD , Pauwels J , Jacobs TB , Chadha NK , Kozak EL , Kozak FK . High frequency of otolaryngology/ENT encounters in Canadian primary care despite low medical undergraduate experiences. Can Med Educ J. 2022;13(1):86‐89. 10.36834/cmej.72328 35291454 PMC8909819

[7] Sun GH , Cain‐Nielsen A , Moloci NM . Who's managing otolaryngologic conditions in the United States? Otolaryngol Head Neck Surg. 2017;157(3):416‐418. 10.1177/0194599817718814 28675093

[8] Urban MJ , Wojcik C , Eggerstedt M , Jagasia AJ . Rural‐urban disparities in otolaryngology: the state of Illinois. Laryngoscope. 2021;131(1):E70‐e75. 10.1002/lary.28652 32249932

[9] Sciscent BY , Chan K , Eberly HW , Goldenberg D , Goyal N . An analysis of the otolaryngology workforce in Pennsylvania. OTO Open. 2024;8(4):e70026. 10.1002/oto2.70026 39386051 PMC11462287

[10] LaCrete F , Ratnapradipa KL , Carlson K , Lyden E , Dowdall JR . Rural‐urban otolaryngologic observational workforce analysis: the state of Nebraska. Laryngoscope Investig Otolaryngol. 2023;8(6):1602‐1606. 10.1002/lio2.1181 PMC10731502 38130258

[11] Winters R , Pou A , Friedlander PA . “medical mission” at home: the needs of rural America in terms of otolaryngology care. J Rural Health. 2011;27(3):297‐301. 10.1111/j.1748-0361.2010.00343.x 21729157

[12] U.S. Census Bureau . 2020 Decennial Census. Demographic and Housing Characteristics, Table P2. Accessed February 20, 2025.

[13] U.S. Census Bureau . 2020 Decennial Census.118th Congressional District Summary File, Table P2. Accessed February 20, 2025.

[14] Centers for Disease Control and Prevention . Health, United States–Physicians. National Center for Health Statistics. Accessed March 20 2025. https://www.cdc.gov/nchs/hus/topics/physicians.htm#references

[15] Mohammed KA , Subramaniam DS , Geneus CJ , et al. Rural‐urban differences in human papillomavirus knowledge and awareness among US adults. Prev Med. 2018;109:39‐43. 10.1016/j.ypmed.2018.01.016 29378268

[16] Doogan NJ , Roberts ME , Wewers ME , et al. A growing geographic disparity: rural and urban cigarette smoking trends in the United States. Prev Med. Nov. 2017;104:79‐85. 10.1016/j.ypmed.2017.03.011 PMC5600673 28315761

[17] Parker MA , Weinberger AH , Eggers EM , Parker ES , Villanti AC . Trends in rural and urban cigarette smoking quit ratios in the US from 2010 to 2020. JAMA Netw Open. 2022;5(8):e2225326. 10.1001/jamanetworkopen.2022.25326 35921112 PMC9350718

[18] Clarke JA , Despotis AM , Ramirez RJ , Zevallos JP , Mazul AL . Head and neck cancer survival disparities by race and rural‐urban context. Cancer Epidemiol Biomarkers Prev. 2020;29(10):1955‐1961. 10.1158/1055-9965.Epi-20-0376 32727721 PMC9073403

[19] Pagedar NA , Davis AB , Sperry SM , Charlton ME , Lynch CF . Population analysis of socioeconomic status and otolaryngologist distribution on head and neck cancer outcomes. Head Neck. 2019;41(4):1046‐1052. 10.1002/hed.25521 30549368 PMC6420379

[20] Eide MJ , Weinstock MA , Clark MA . The association of physician‐specialty density and melanoma prognosis in the United States, 1988 to 1993. J Am Acad Dermatol. 2009;60(1):51‐58. 10.1016/j.jaad.2008.08.040 18937998 PMC2636622

[21] Odisho AY , Cooperberg MR , Fradet V , Ahmad AE , Carroll PR . Urologist density and county‐level urologic cancer mortality. J Clin Oncol. 2010;28(15):2499‐2504. 10.1200/jco.2009.26.9597 20406931 PMC2881728

[22] Goodman DC , Fisher ES , Little GA , Stukel TA , Chang CH , Schoendorf KS . The relation between the availability of neonatal intensive care and neonatal mortality. N Engl J Med. 2002;346(20):1538‐1544. 10.1056/NEJMoa011921 12015393

[23] Wang W , Han S , Yao Z , et al. A study of epidemiologic and recurrence factors of oral cancer. J Oral Maxillofac Surg. 2012;70(9):2205‐2210. 10.1016/j.joms.2011.09.040 22209101

[24] Zhang H , Dziegielewski PT , Jean Nguyen TT , et al. The effects of geography on survival in patients with oral cavity squamous cell carcinoma. Oral Oncol. 2015;51(6):578‐585. 10.1016/j.oraloncology.2015.03.012 25865551

[25] Adelman AE , Khan A , Armosh K , Lanza J , Lobo BC . Current state of Florida's otolaryngology workforce. Laryngoscope. 2026;136(1):168‐174. 10.1002/lary.70010 40751510

[26] Shah D , Long WE , Eskandari S , Bozzone A , Parada SA . Geospatial analysis of orthopedic workforce distribution across Georgia. Cureus. 2025;17(10):e95588. 10.7759/cureus.95588 41322886 PMC12661093

[27] Eskandari S , Shah D , Long WE , Savage NM , Islam S , Healy WJ . Distribution of the Georgia pulmonology workforce: a rural‐urban analysis. South Med J. 2026;119(3):146‐151. 10.14423/SMJ.0000000000001946 42000194

[28] U.S. Centers for Medicare & Medicaid Services . Medicare Physician & Other Practitioners by Provider and Service. Data.CMS.Gov. 2025. https://data.cms.gov/provider-summary-by-type-of-service/medicare-physician-other-practitioners/medicare-physician-other-practitioners-by-provider-and-service

[29] Georgia Composite Medical Board . Order a Copy of the Medical Board Database. Georgia.gov. Accessed February 20, 2025. https://medicalboard.georgia.gov/consumer-resources/order-copy-medical-board-database

[30] American Academy of Otolaryngology–Head and Neck Surgery . Find an ENT. ENT Health. Accessed February 20, 2025. www.enthealth.org/find-ent.

[31] U.S. Census Bureau . 2020 Decennial Census. Demographic and Housing Characteristics, Table P1. Accessed February 20, 2025.

[32] U.S. Census Bureau . 2023 American Community Survey. ACS 5‐Year Estimates, Table B19013. Accessed February 20, 2025.

[33] United States Department of Agriculture Economic Research Service. Rural‐Urban Continuum Codes. Accessed February 20, 2025. https://www.ers.usda.gov/data-products/rural-urban-continuum-codes

[34] Griffiths E . Incidence of ENT problems in general practice. J R Soc Med. 1979;72(10):740‐742. 10.1177/014107687907201008 552431 PMC1437201

[35] Hannaford PC , Simpson JA , Bisset AF , Davis A , McKerrow W , Mills R . The prevalence of ear, nose and throat problems in the community: results from a national cross‐sectional postal survey in Scotland. Fam Pract. 2005;22(3):227‐233. 10.1093/fampra/cmi004 15772117

[36] Urban MJ , Shimomura A , Shah S , Losenegger T , Westrick J , Jagasia AA . Rural otolaryngology care disparities: a scoping review. Otolaryngol Head Neck Surg. 2022;166(6):1219‐1227. 10.1177/01945998211068822 35015580

[37] Vickery TW , Weterings R , Cabrera‐Muffly C . Geographic distribution of otolaryngologists in the United States. Ear Nose Throat J. 2016;95(6):218‐223. 27304439

[38] Miller RH , McCrary HC , Gurgel RK . Assessing trends in fellowship training among otolaryngology residents: a national survey study. Otolaryngol Head Neck Surg. 2021;165(5):655‐661. 10.1177/0194599821994477 33618575

[39] Miller RH , Gurgel RK , McCrary HC . Graduating otolaryngology residents’ ideal practice expectations: a longitudinal analysis. Otolaryngol Head Neck Surg. 2022;167(3):472‐478. 10.1177/01945998211069505 34982583

[40] Arcury TA , Preisser JS , Gesler WM , Powers JM . Access to transportation and health care utilization in a rural region. J Rural Health. 2005;21(1):31‐38. 10.1111/j.1748-0361.2005.tb00059.x 15667007

[41] U.S Department of Transportation . The Critical Role of Rural Communities. Accessed March 20, 2025. https://www.transportation.gov/rural/grant-toolkit/critical-role-rural-communities

[42] Salemink K , Strijker D , Bosworth G . Rural development in the digital age: a systematic literature review on unequal ICT availability, adoption, and use in rural areas. J Rural Stud. 2017;54:360‐371. 10.1016/j.jrurstud.2015.09.001

[43] Arredondo K , Touchett HN , Khan S , Vincenti M , Watts BV . Current programs and incentives to overcome rural physician shortages in the United States: a narrative review. J Gen Intern Med. 2023;38(Suppl 3):916‐922. 10.1007/s11606-023-08122-6 37340266 PMC10356718

